# Cardiac Magnetic Resonance versus Single-Photon Emission Computed Tomography for Detecting Coronary Artery Disease and Myocardial Ischemia: Comparison with Coronary Angiography

**DOI:** 10.3390/diagnostics10040190

**Published:** 2020-03-29

**Authors:** Fotios Laspas, Theodoros Pipikos, Emmanouil Karatzis, Nikolaos Georgakopoulos, Vasileios Prassopoulos, John Andreou, Lia A. Moulopoulos, Achilleas Chatziioannou, Peter G. Danias

**Affiliations:** 1 Department of CT-MRI, “Hygeia” Hospital, 15123 Athens, Greece; j.andreou1@gmail.com; 2 Department of Nuclear Medicine, “Hygeia” Hospital, 15123 Athens, Greece; theodpipikos@gmail.com (T.P.); VPrasopoulos@hygeia.gr (V.P.); 3 Department of Cardiac MR, “Hygeia” Hospital, 15123 Athens, Greece; mkaratzis@hygeia.gr (E.K.); pdanias@hygeia.gr (P.G.D.); 4 Department of Intervetional Cardiology, “Hygeia” Hospital, 15123 Athens, Greece; NGeorgakopoulos@hygeia.gr; 5 First Department of Radiology, University of Athens School of Medicine, “Areteion” Hospital, 11528 Athens, Greece; lmoulop@med.uoa.gr (L.A.M.); achatzi@med.uoa.gr (A.C.); 6 Department of Medicine, Tufts University Medical School, Boston, MA 02111, USA

**Keywords:** coronary artery disease, single-photon emission computed tomography, cardiac magnetic resonance, myocardial perfusion

## Abstract

Background: This study aimed to compare the diagnostic accuracy of stress single-photon emission computed tomography (SPECT) and stress cardiac magnetic resonance (CMR) for the assessment of coronary artery disease (CAD) in the same patients, using coronary angiography as the reference standard. Methods: Thirty patients with known or suspected CAD who were referred for exercise SPECT myocardial perfusion imaging (MPI) for the evaluation of myocardial ischemia underwent stress CMR MPI and computed tomography coronary angiography (CTCA) or selective coronary angiography (SCA). The data from the two stress modalities were compared against the data from angiography. Results: In our study population, 30% of the recruited subjects had significant CAD. The CMR sensitivity for the detection of significant CAD and/or myocardial ischemia was 89% and specificity was 76%. For SPECT, the corresponding sensitivity was 78% and specificity was 52%. The negative predictive value was 92% for CMR and 83% for SPECT. The receiver-operating characteristic (ROC) analysis evaluating the presence of significant CAD, CMR (area under the curve (AUC) 0.78) outperformed SPECT (AUC 0.59) (*p* < 0.01). The ROC analysis evaluating the presence of myocardial ischemia was also in favor of CMR (AUC 0.82) versus SPECT (AUC 0.67) (*p* < 0.01). Conclusions: CMR has high diagnostic accuracy for the detection of CAD and stress-induced ischemia and appears to outperform SPECT. CMR may thus be the preferred noninvasive imaging modality to assess patients with known or suspected CAD.

## 1. Introduction

Coronary artery disease (CAD) is a major public health concern and a leading cause of death in the western world [1]. The appropriate evaluation of patients with known or suspected CAD has traditionally rested on two pillars: morphologic assessment for coronary artery stenosis and functional evaluation for determining the downstream hemodynamic significance of lesions. Various imaging methods are used to assess the presence and extent of CAD and myocardial ischemia. Anatomical imaging techniques, such as computed tomography coronary angiography (CTCA) or invasive selective coronary angiography (SCA), allow the direct assessment of coronary artery integrity; however, the evaluation of myocardial ischemia involves more than determining coronary anatomy. Functional imaging techniques, such as stress single-photon emission computed tomography (SPECT), positron emission tomography (PET), stress echocardiography and stress cardiac magnetic resonance (CMR), assess the hemodynamic consequences of CAD by detecting stress or vasodilator-related changes in myocardial perfusion and/or wall motion abnormalities [2]. The presence and extent of myocardial ischemia determine a patient’s risk for subsequent cardiovascular events and death, and assist in the selection of optimal therapy [3].

Functional imaging based on myocardial perfusion is more sensitive but less specific than functional imaging based on ventricular wall motion [4]. Presently, stress SPECT myocardial perfusion imaging (MPI) is the most widely used clinical tool to assess myocardial perfusion. In the last decade, CMR has evolved as an alternative technique to assess myocardial perfusion at stress (maximal vasodilation) and rest. The substantial potential advantages of CMR over SPECT include the lack of exposure to ionizing radiation, high spatial resolution and the ability to assess morphology, function, tissue characterization and coronary artery integrity with a single examination (“one-stop shop”) [5,6]. Previous studies [7,8,9] have assessed the diagnostic accuracy of stress CMR MPI to diagnose CAD. Other studies [5,10,11] have performed a head-to-head comparison of stress CMR vs. SPECT MRI. Among these, the CE-MARC [5] study is, to date, the largest single-center clinical trial where CMR was compared against SPECT nuclear imaging, using coronary angiography as the reference standard. This study established the clinical utility of stress CMR. A substudy of CE-MARC [12], and one more study from the same senior investigator [13], have addressed the diagnostic utility of stress CMR for the detection of stress-induced myocardial ischemia, indicating a need for more data in this direction.

The aim of this study was to compare the diagnostic accuracy of stress SPECT and CMR MPI for the assessment of CAD and myocardial ischemia in the same patients with known or suspected CAD, using coronary angiography as the reference standard.

## 2. Materials and Methods 

### 2.1. Study Design and Patient Population

Patients were recruited between September 2017 and September 2018 at Hygeia Hospital, a tertiary health care facility in Athens, Greece. The study was approved by the “Hygeia Hospital Scientific Council-Ethics Committee” (598/15-4-2017) and all subjects signed informed consent prior to participation. Consecutive patients with known or suspected CAD that were referred to the hospital for a stress SPECT MPI evaluation of myocardial ischemia were screened. In this preliminary investigation, thirty patients that underwent a diagnostic exercise stress SPECT (i.e., achieved > 85% of age-predicted maximal heart rate) and had no contraindications for CMR were prospectively invited to participate. All participants, after the stress SPECT MPI, underwent adenosine stress CMR MPI and CTCA, or SCA if the patient’s cardiologist decided to proceed to an invasive approach based on the SPECT MPI and/or clinical findings. Given the excellent negative predictive value of CTCA in excluding significant CAD, in patients with negative CTCA no further investigation was performed, as per standard clinical practice and guideline recommendations [14,15]. The maximum interval between all examinations was 2 weeks. Exclusion criteria were atrial fibrillation, contraindication to CMR (e.g., pacemaker, defibrillator) and contraindication to gadolinium (glomerular filtration rate < 30 mL/min) or adenosine (severe asthma, severe conduction abnormalities). The SPECT and CMR MPI and coronary angiography (CTCA and SCA) were evaluated by investigators trained and board-certified in nuclear medicine, radiology and/or cardiology with at least 10 years of experience of using the corresponding modalities.

### 2.2. SPECT MPI

Stress SPECT MPI studies were performed with a one-day 99m-Tc-tetrofosmin stress/rest protocol using a hybrid SPECT/CT scanner (BrightView XCT, Philips Medical Systems, The Netherlands). All stress SPECT studies by inclusion criteria were performed by graded exercise on a treadmill using a standard Bruce protocol, and all patients achieved the required exercise threshold. At peak exercise, 8 mCi (296 MBq) of 99m-Tc-tetrofosmin were injected intravenously. Post-stress images were acquired 5-10 minutes after completion of the stress protocol. Two and a half hours after completion of the stress image acquisition, 20 mCi (740 MBq) 99m-Tc-tetrofosmin were injected for the rest study and images were acquired 45 min later. Electrocardiogram (ECG)-gated images were acquired in supine position for both stress and rest. Attenuation correction was applied to all SPECT examinations using a CT-based attenuation map in order to overcome artefacts due to photon absorption, to enhance image quality and improve diagnostic accuracy [16]. All data were transferred to a dedicated workstation for analysis.

The images of stress and rest SPECT perfusion scans were evaluated visually using a 17-segment AHA/ACC model [17]. Each segment was scored on a 5-point scale: 0 = normal, 1 = mild reduction in tracer uptake, 2 = moderate reduction in uptake, 3 = significant reduction in uptake and 4 = absence of uptake. Ventricular volumes (end diastolic and end systolic) were calculated and wall motion abnormalities were assessed, and diagnosis was made on the basis of all available SPECT data.

### 2.3. CMR MPI

All CMR MPI examinations were performed on a 1.5T clinical MRI scanner (Achieva, Philips Medical Systems, The Netherlands) equipped with a 5-element cardiac phase-array coil. MR images were obtained with ECG gating. The CMR protocol included anatomic imaging, ventricular function cine imaging, stress and rest perfusion study and late gadolinium enhancement (LGE) technique. For the vasodilator stress imaging, adenosine was infused at a rate of 0.14 mg/Kg/min through a peripheral intravenous line for 4 min under continuous heart rate, blood pressure and pulse oximetry monitoring. Perfusion stress sequence was then applied during a bolus injection of 0.075 mmol/kg of gadolinium (Dotarem, Guerbet, France) through a separate IV line in the other arm. Three short-axis images (base, mid, and apex) were obtained using a T1-weighted saturation-recovery balanced turbo field-echo pulse sequence and obtaining images at every heartbeat (voxel size 2.5–3.3 mm). Rest perfusion images were obtained at least 10 min after stress perfusion at the same locations and using the same contrast dose. Ventricular function imaging was performed between the stress and rest acquisitions using a balanced cine, steady-state, free precession pulse sequence. After the rest study, a final injection of 0.05 mmol/kg of gadolinium was given, reaching the total gadolinium dose of 0.2 mmol/kg. Finally, a late enhancement study with a T1-weighted segmented inversion–recovery gradient echo pulse sequence was performed in the short axis and four-chamber orientation. All CMR imaging was performed during end-expiration breath-holding.

CMR MPI was visually interpreted. Sixteen segments of the 17-segment AHA/ACC model (excluding apical segment #17) were scored using a 5-point scale: 0 = normal, 1 = possible defect, 2 = mild defect, 3 = moderate and 4 = significant defect. In general, the presence of myocardial perfusion defects, hyperintense areas with a subendocardial ischemic pattern, on LGE images, and any evidence of segmental wall motion abnormality (hypokinesia, akinesia or dyskinesia), was considered indicative of CAD. Ischemia was identified as a stress-induced reversible defect without matching high-signal intensity on the LGE images.

### 2.4. Coronary Angiography

All coronary angiographies were performed after SPECT and CMR. The CTCA examinations were performed on a dual-source 128-detector CT scanner (Somatom Definition, Siemens Medical Solutions, Forchheim, Germany), achieving diagnostic image quality in all cases. All SCA examinations were performed by an experienced interventional clinical cardiologist in multiple projections, as per standard guidelines. For both anatomical methods (CTCA and SCA), hemodynamically significant stenosis was defined as ≥ 70% luminal narrowing of a coronary artery measuring ≥ 2 mm in diameter, or ≥ 50% of the left main coronary artery.

### 2.5. Comparison of the Procedures

For the comparison of CMR to SPECT MPI, the summed stress score (SSS), summed rest score (SRS) and summed difference score (SDS) were calculated. The SSS and SRS are obtained by adding the scores of the segments that were analyzed and scored during a stress and a rest study, respectively. The SDS is the difference between SSS and SRS. The SSS is an index that incorporates the extent and severity of perfusion defects and is associated with total coronary artery disease burden, indicating an ischemic and infarcted myocardium. When SSS is combined with the SRS, the difference (SDS) incorporates the degree of defect reversibility, reflecting the amount of stress-induced ischemia. An SSS ≥ 4 was considered abnormal, indicating significant coronary heart disease, and an SDS ≥ 2 was deemed abnormal, reflecting reversible ischemia.

The sensitivity and specificity of SPECT and CMR MPI were calculated and receiver-operating characteristic (ROC) analysis was performed.

### 2.6. Statistical Analysis

Statistical analysis was performed using SPSS software (version 22.0). Sensitivity, specificity, negative and positive predictive values were calculated for SPECT and CMR MPI and were compared using a Mc Nemar test. ROC curves were used in order to estimate the discriminative ability of SSS and SDS. The overall performance of the ROC analysis was quantified by computing the area under the curve (AUC). An area of 1.0 indicated perfect performance, while 0.5 indicated a performance that could not be differentiated from chance. All *p* values reported are two-tailed. Statistical significance was set at 0.05.

## 3. Results

**Figure 1:**
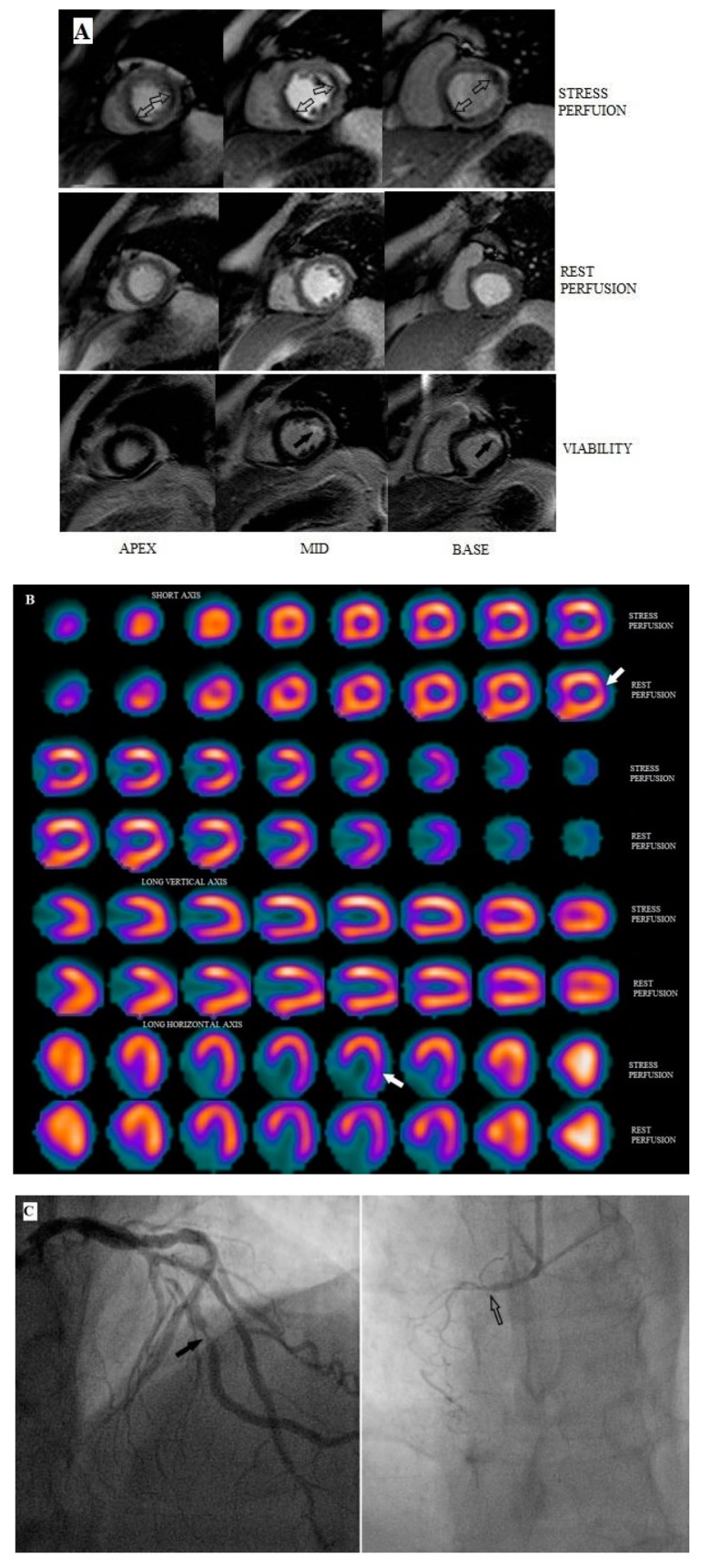
Representative images from a 72 year-old male patient with chest pain. (**A**) Stress-rest cardiac magnetic resonance (CMR) images show stress-induced perfusion defects (ischemia) in the lateral-anterolateral and inferoseptal left ventricular walls of the basal, mid and apical slices (open arrows) and subendocardial late gandolinium enhancement (LGE) (non-transmural infarction) in the anterolateral wall at the basal and mid-ventricular slices (black arrows). (**B**) single-photon emission computed tomography (SPECT) shows a partially reversible defect (white arrows) at the lateral and anterolateral left ventricular walls, but fails to demonstrate the inferoseptal ischemia. (**C**) SCA demonstrates a significant (>70%) luminal stenosis (left panel, black arrow) at the obtuse marginal branch and occlusion of the right coronary artery (right panel, open arrow) confirming the CMR findings.

Thirty patients (24 men and six women, age range 46–75 years; mean age 65.3 years) were enrolled in the study and underwent stress SPECT and CMR MPI and coronary angiography (CTCA or SCA). The clinical characteristics of the study population are presented in Table 1. Diagnostic images were successfully obtained for each patient by all modalities. Representative example images are shown in Figure 1.

SCA was performed on 19 participants for whom there was a high clinical suspicion for significant CAD. CTCA was performed on 11 participants with a low to intermediate clinical probability of CAD. On SCA, ≥ 70% stenosis of at least one coronary artery was observed in nine of 19 patients (47.4%). None of the patients who underwent CTCA were found to have severe stenosis. The overall prevalence of significant CAD was 30%.

**Figure 2:**
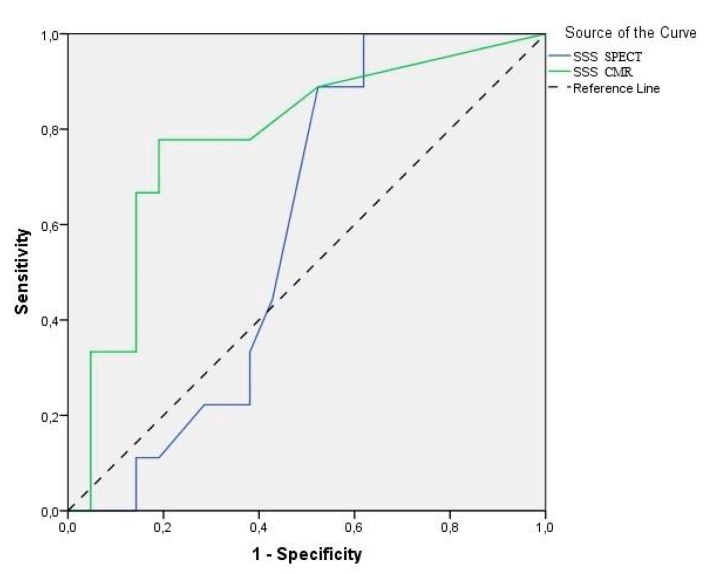
Receiver-operating characteristic (ROC) curves of summed stress score (SSS) for stress CMR and SPECT myocardial perfusion imaging (MPI) in detecting significant angiographic CAD (≥70% stenosis). The area under curve (AUC) was 0.78 and 0.59 for CMR and SPECT, respectively. Only CMR detected CAD had the ability to predict positive findings on coronary angiography (*p* < 0.01).

**Figure 3:**
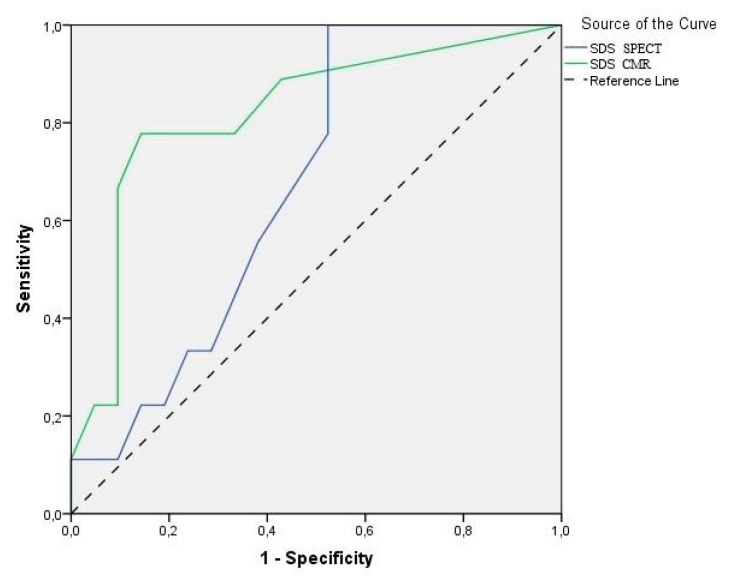
ROC curves of SDS (i.e., ability to detect stress-induced myocardial ischemia) for MPI by stress CMR and SPECT, as determined by coronary artery angiography (≥70% stenosis). The AUC was 0.82 for CMR and 0.67 for SPECT. Only CMR-determined ischemia had the ability to predict positive findings on coronary angiography (*p* < 0.01).

For CMR MPI, the sensitivity and specificity for identifying patients with obstructive heart disease were 88.9% and 76.2%, respectively. For SPECT MPI the corresponding sensitivity and specificity were 77.8% and 52.4%, respectively. The negative predictive value for detecting significant CAD was 92.3% for CMR and 83.3% for SPECT. There was no statistically significant difference regarding the aforementioned values between CMR and SPECT MPI (*p* > 0.05 for all comparisons). However, on ROC analysis generated using SSS, the MPI by CMR (AUC 0.78, 95% CI 0.59–0.97) outperformed that by SPECT (AUC 0.59, 95% CI 0.39–0.79) in predicting the presence of severe stenosis on coronary angiography (*p* < 0.01) (Figure 2). Moreover, the ROC analysis calculated from stress and rest perfusion images using SDS was in favor of CMR (AUC 0.82, 95% CI 0.65–0.99) versus SPECT (AUC 0.67, 95% CI 0.48–0.86) and showed a significant predictive ability for positive coronary angiography only for CMR (*p* < 0.01) (Figure 3).

## 4. Discussion

In this prospective study involving a small but very well-defined group of patients, we compared head-to-head stress CMR and SPECT MPI for the detection of significant CAD and stress-induced ischemia, using coronary angiography as the reference standard. We found that MPI by stress CMR outperformed SPECT in predicting both the presence of CAD and ischemia on ROC analyses, although both techniques had comparable high values for sensitivity and specificity.

A number of investigations, including single-center [7,8,9] and multi-center studies [10,11], have validated the high accuracy of stress CMR MPI to detect CAD. As stress CMR evolved to maturity, comparison with stress SPECT MRI was performed in either original investigations [5,11] or meta-analyses [18,19]. Most published data comparing the two techniques assessed the ability of stress CMR vs. SPECT MPI to detect significant CAD, as determined by the SSS. 

Two multicenter prospective trials have compared the accuracy of CMR and SPECT for the detection of CAD, Magnetic Resonance Imaging for Myocardial Perfusion Assessment in Coronary artery disease (MR-IMPACT I) [10] and MR-IMPACT II [11]. The first study concentrated primarily on identifying the optimal contrast dose for the stress CMR studies, while the second one concentrated on comparing the two modalities for the detection of CAD, using any perfusion abnormality at stress or test as an indication of CAD. MR-IMPACT II recruited 533 patients in 33 European and U.S. centers, and gated-SPECT was not performed in all patients. The sensitivity of first-pass perfusion CMR in detecting angiographic significant CAD was superior to SPECT (75% vs. 59% respectively). However, the specificity of CMR perfusion was lower than SPECT (59% vs. 72% respectively), likely because both highly experienced and less experienced centers participated. Our study had a similar sensitivity and better specificity for stress CMR, compared with the MR-IMPACT II study.

Greenwood et al. published the largest prospective trial to date, with 752 patients, comparing stress CMR and SPECT for the diagnosis of angiographically proven CAD (Clinical Evaluation of MAgnetic Resonance imaging in Coronary heart disease (CE-MARC)) [5]. The differences between the sensitivity and negative predictive value of CMR and SPECT were significantly in favor of CMR (*p* < 0.0001 for both), but specificity and positive predictive value did not differ significantly (*p* = 0.916 and *p* = 0.061, respectively). This trial established the high diagnostic accuracy of CMR imaging in CAD and suggested its superiority over SPECT. While CE-MARC excluded patients with CABG (one of the study limitations quoted in the publication), CABG patients were included in our study for a more realistic clinical setting representation. On the other hand, in CE-MARC all patients were prospectively scheduled to undergo CMR, SPECT and SCA at the time of recruitment, while in our study SPECT was the first examination. These differences in study design, along with the smaller sample size in our investigation, likely explain the higher specificity of stress SPECT in the CE-MARC (82.6% vs. 52.4% in our study), despite our use both ECG-gating and attenuation correction in all SPECT studies. The results of CMR in our study were in keeping with those of CE-MARC (sensitivity 88.9% vs. 86.5% and specificity 76.2% vs. 83.4%). Finally, in CE-MARC ROC curves were calculated from the stress perfusion images only, and in this analysis our results are similar to that of CE-MARC (AUC 0.82 vs. 0.89).

Two meta-analyses have confirmed the high accuracy of stress CMR in diagnosing significant CAD, using quantitative coronary angiography as the reference standard [18,19]. In the first, de Jong and colleagues combined data from 4293 patients in 41 different studies to demonstrate an overall patient-based sensitivity of 91% and specificity of 80% for stress CMR, and a sensitivity of 83% and specificity of 77% for SPECT [18]. CMR had a better sensitivity than SPECT (*p* = 0.03), but specificity was similar. The second meta-analysis of 166 articles published between the period 1990 and 2010 evaluated the diagnostic performance of noninvasive perfusion imaging modalities for the detection of obstructive CAD [19]. The patient-based analysis per imaging modality demonstrated a pooled sensitivity of 89% and 88% for CMR and SPECT, respectively, and a pooled specificity of 76% and 61% for CMR and SPECT, respectively. The results of this meta-analysis showed similar pooled sensitivities for the two modalities and significant differences in pooled specificities (better for stress CMR).

The data in the literature are scarce regarding the ability of stress CMR to identify stress-induced myocardial ischemia. The identification of ischemia is clinically pertinent, as its presence is associated with significantly increased major cardiovascular events [20,21]. A substudy of CE-MARC [12] including 106 subjects addressed the assessment of ischemic burden by CMR and SPECT MPI, comparing the SDS in both modalities against coronary angiography. In that report, CMR measured significantly more ischemia than SPECT did. Another research from the same senior investigator has also addressed the same question in a group of 46 patients using a three-dimensional CMR imaging sequence [13]. In this study, 33 patients had angiographic confirmation and sensitivity/specificity were similar for CMR and SPECT MPI. Our study results are in line with those from the CE-MARK substudy, as our findings also support that CMR MPI detects significantly more ischemia than SPECT MPI.

In recent years, fractional flow reserve (FFR) has become the preferred reference standard for studies assessing stress myocardial perfusion. Thirty-seven studies reporting on 2048 patients were included in a meta-analysis comparing the accuracy of myocardial perfusion modalities for the diagnosis of haemodynamically significant CAD [22]. For CMR, the pooled estimates of diagnostic accuracy (including sensitivity and specificity) were substantially higher than SPECT. Our study did not include FFR measurements. Although this could be considered as a limitation, it would be unethical to perform FFR in patients with either a low likelihood of CAD, for whom CT coronary angiography was chosen to define coronary artery anatomy, or those with no or minimal CAD on conventional angiography.

The major limitation of our study is the rather small number of patients included, accounting for a relatively wide confidence interval in the assessment of specificity and sensitivity. This small number does not allow for further investigation of differences between subgroups, such as those with a high clinical suspicion for CAD that underwent SCA (roughly two thirds of our patients) or with documented significant CAD (roughly one third of our patients). Thus, the results of our study should be viewed only as preliminary data, suggestive of the potential of better diagnostic performance of CMR versus SPECT MPI to assess CAD and myocardial ischemia. Furthermore, the stress protocols differed between SPECT (exercise) and CMR (vasodilation). However, the accuracy of adenosine stress SPECT is similar to that of exercise stress SPECT [23] and the difference between stress techniques is not expected to have affected the findings.

## 5. Conclusions

Our single-center study in a well-defined, small number of patients undergoing stress SPECT and CMR MPI versus coronary angiography supports that stress CMR MPI has high diagnostic accuracy for the detection of CAD and stress-induced ischemia and compares favorably to stress SPECT MPI. As CMR offers a comprehensive noninvasive and radiation-free assessment of left ventricular function, myocardial perfusion, wall motion and myocardial viability in a single imaging test, it may be the preferred noninvasive imaging modality to assess patients with known or suspected CAD, and to guide clinical decisions in this patient population.

## Figures and Tables

**Table 1:** Patient characteristics.

Patient Characteristics	N (%)
Patients enrolled	30
Male gender	24 (80%)
Mean age (Age range) (years)	65.3 (46–75)
Mean Body Mass Index (kg/m^2^)	28.4
Previous stent placement	5 (17%)
Previous coronary artery bypass graft surgery (CABG)	2 (7%)
Family history of coronary artery disease (CAD)	14 (47%)
Current Smokers	5 (17%)
Hypertension	17 (57%)
Hypercholesterolemia	16 (53%)
Diabetes mellitus	5 (17%)
≥70% stenosis by selective coronary angiography (SCA)	9 (30%)
